# Supplementary material for: HDAC1/2-mediated repression of Wnt receptor expression orients asymmetric division polarity in Caenorhabditis elegans
Source: Development. 2026 Sep 3;153(17):dev205658. doi: 10.1242/dev.205658 (PMC13615694; doi:10.1242/dev.205658)
Supplement: Supplementary file 1 [file develop-153-205658-s1.pdf]

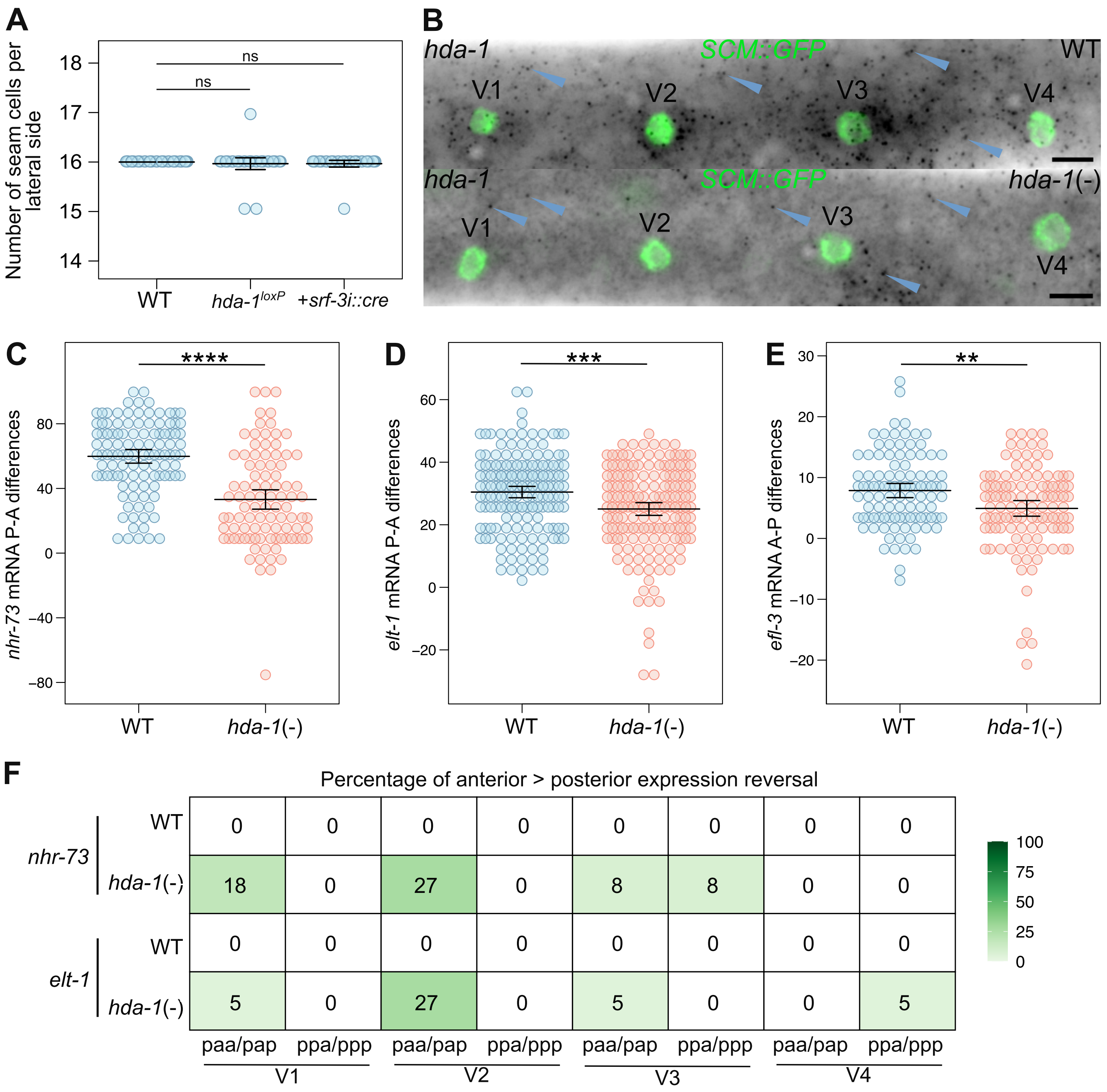

Development • Supplementary information

Fig. S1. Generation of tissue-specific *hda-1* mutants. (A) Seam cell counts in wild-type, animals with *hda-1* locus floxed and animals expressing the CRE recombinase in the seam cells (+*srf-3::cre*). n = 30 animals per condition (Krustal-Wallis test). **(B)** Representative images of *hda-1* smFISH at late L1 stage in wild-type (WT) and in *hda- 1* mutant animals (*hda-1*(-)). *hda-1*(-) animals don't show *hda-1* mRNA spots in the seam cells. Blue arrowheads point to *hda-1* mRNA spots in the hypodermis. Seam cell nuclei are labelled using *SCM::GFP* and scale bars are 5 μm. **(C-E)** Quantification of anterior-posterior mRNA difference scores from Fig. 3 for *nhr-73* (C), *elt-1* (D) and *efl- 3* (E). For C and D, difference scores were calculated as posterior – anterior whereas for E they were anterior – posterior. **(F)** Heatmap showing the percentage of *nhr-73* and *elt-1* sister cell pairs displaying anterior > posterior expression reversal following the L2 asymmetric division in WT and in *hda-1*(-) animals. $10 \leq n \leq 22$ cell pairs per condition. Expression frequencies significantly differed between WT and *hda-1* mutants in paa-pap cell pairs (Fisher's exact test). Error bars in A, C-E show the mean ± 95% confidence intervals and in C-E stars represent: **** p<0.0001, *** p<0.001, ** p<0.01 with a Mann-Whitney test.

Development • Supplementary information

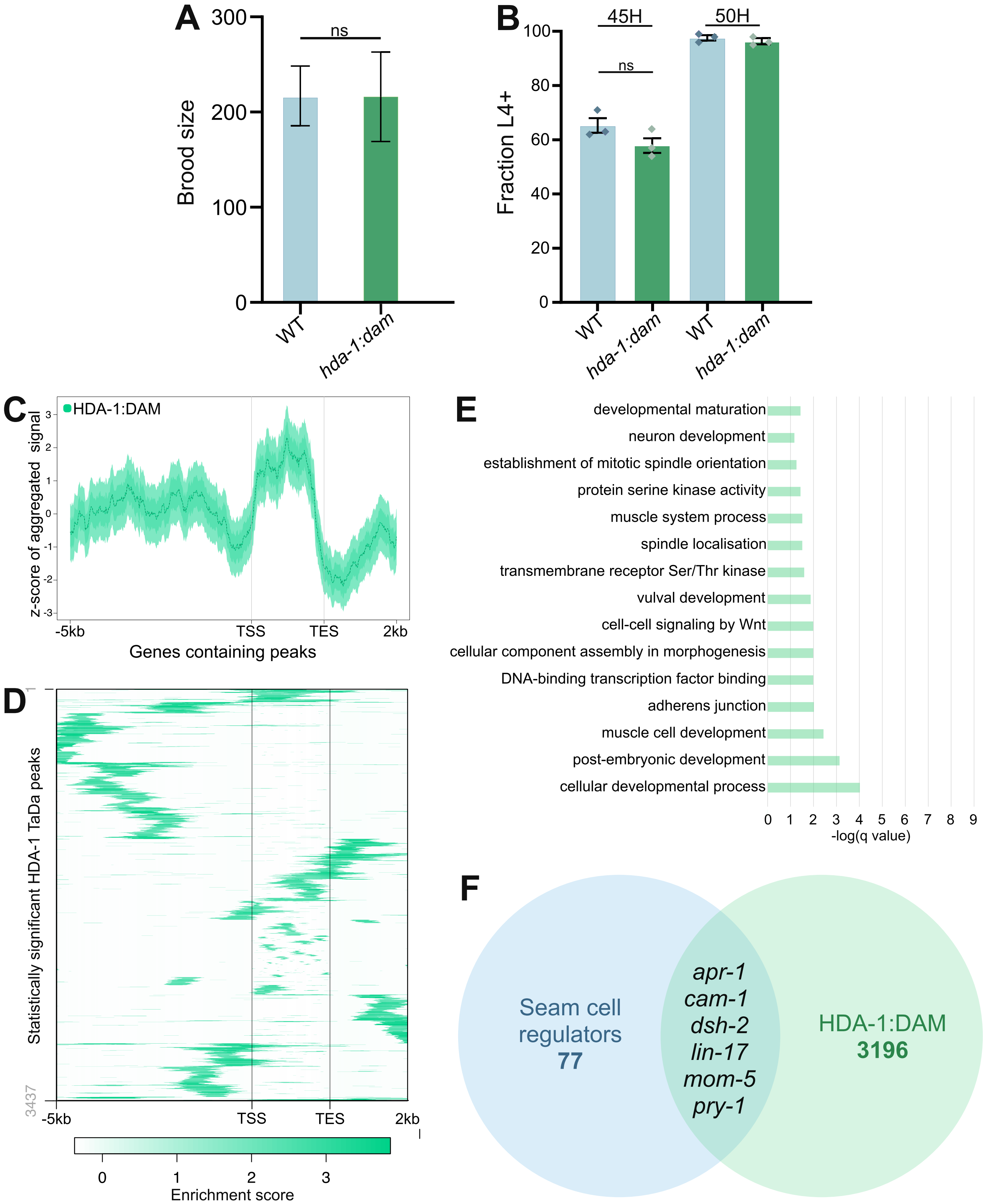

Development • Supplementary information

Fig. S2. TaDa analysis of HDA-1 binding. (A) Quantification of brood size for animals containing single-copy *hda-1:dam* transgene shows no differences compared to control animals, n = 10 animals. **(B)** Quantification of developmental pace by measuring the fraction of animals at or beyond the L4 stage (+L4 stage) at 45h and 50h post egg laying for animals containing a single-copy *hda-1:dam* transgene. Error bars indicate the standard error of proportion, and the bar plot corresponds to the average proportion across three biological replicates. The three datapoints represent values for the three individual replicates. No statistically significant difference in the average proportion of +L4 stage was observed in *hda-1:dam* animals compared to WT with a Chi-square test. **(C)** Aggregation plots of profiling data generated by HDA-1 TaDa showing average enrichment scores in 10-bp bins for regions of equal length across all genes containing statistically significant peaks. Plots show 6 kb upstream of the transcription start site (TSS) and 2 kb downstream of the transcription end site (TES). Gene bodies are condensed into a 2 kb pseudo-length. Shaded areas represent 95% confidence intervals. **(D)** Heatmaps representing the hierarchically clustered localisation and enrichment score of all statistically significant peaks within 6 kb upstream and 2 kb downstream of genes containing peaks. **(E)** Plots of selected significantly enriched terms from GO-term analysis for HDA-1 TaDa. **(F)** Venn diagram highlighting the 6 genes we focused on in this study which are part of the overlap (29 genes) between HDA-1 putative target genes identified with TaDa (green) and a list of 77 genes known to participate in the regulation of seam cell development based on the literature (blue). For a complete list of genes see Table S2.

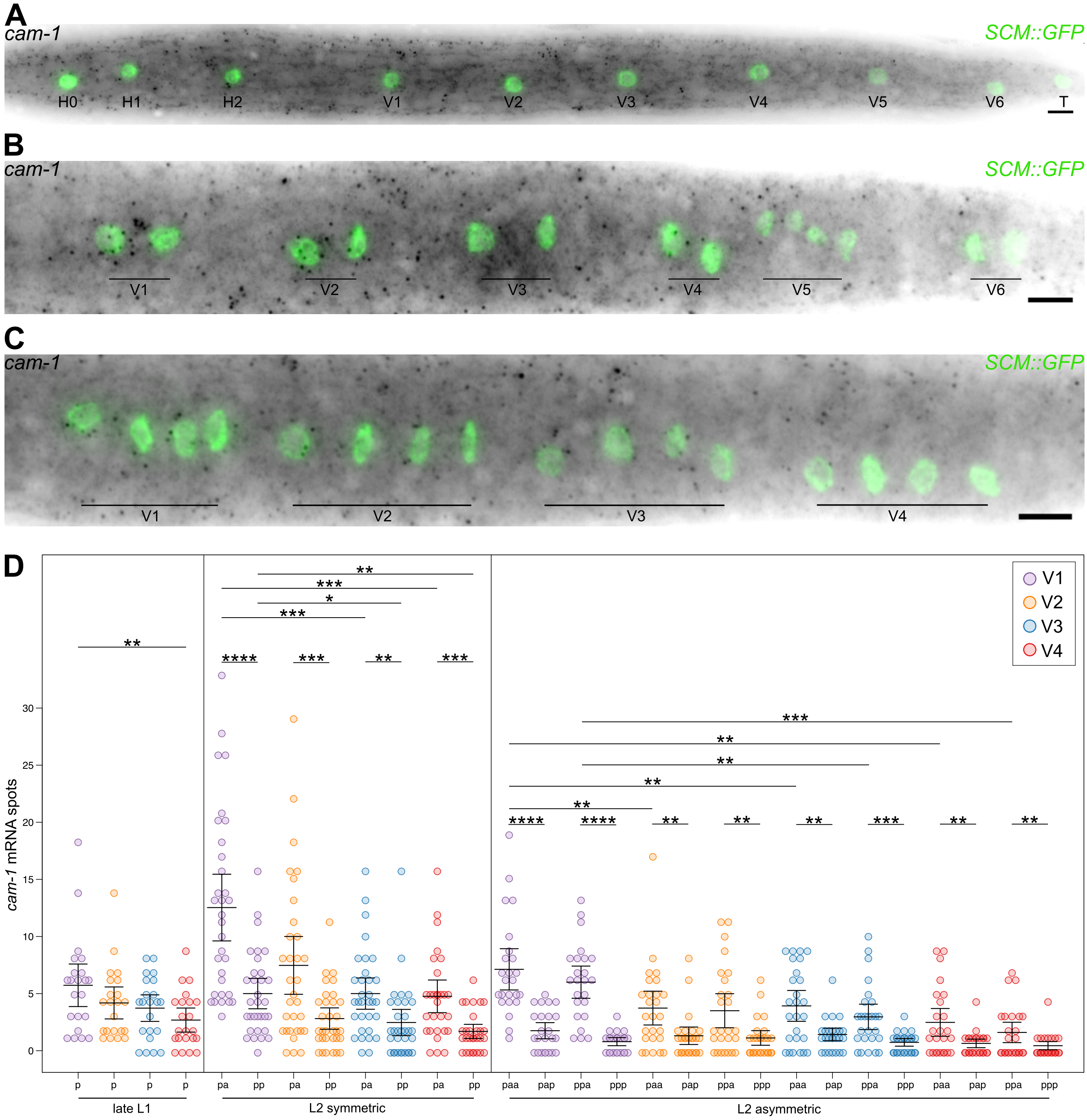

Fig. S3. Detailed analysis of *cam-1* expression in wild-type epidermis. (A-C) Representative *cam-1* smFISH images at late L1 (A), and following the L2 symmetric (B) and L2 asymmetric (C) divisions. Seam cell nuclei are labelled with *SCM::GFP* and scale bars are 5µm. **(D)** Quantification of *cam-1* mRNA spots in V1-V4 seam cells before (p) and after the L2 symmetric division (pa, pp), and following the L2 asymmetric division (paa, pap, ppa, ppp). Across lineage comparisons were analysed using Friedman tests followed by post hoc Wilcoxon signed-rank tests. Within lineage anterior/posterior comparisons were analysed using paired t-tests or Wilcoxon signed-rank tests, according to normality testing. For clarity, only statistically significant comparisons are indicated and stars represent: **** $p<0.0001$, *** $p<0.001$, ** $p<0.01$ and * $p<0.05$; $22 \leq n \leq 32$ cells per condition. Error bars in D show the mean ± 95% confidence intervals.

Development • Supplementary information

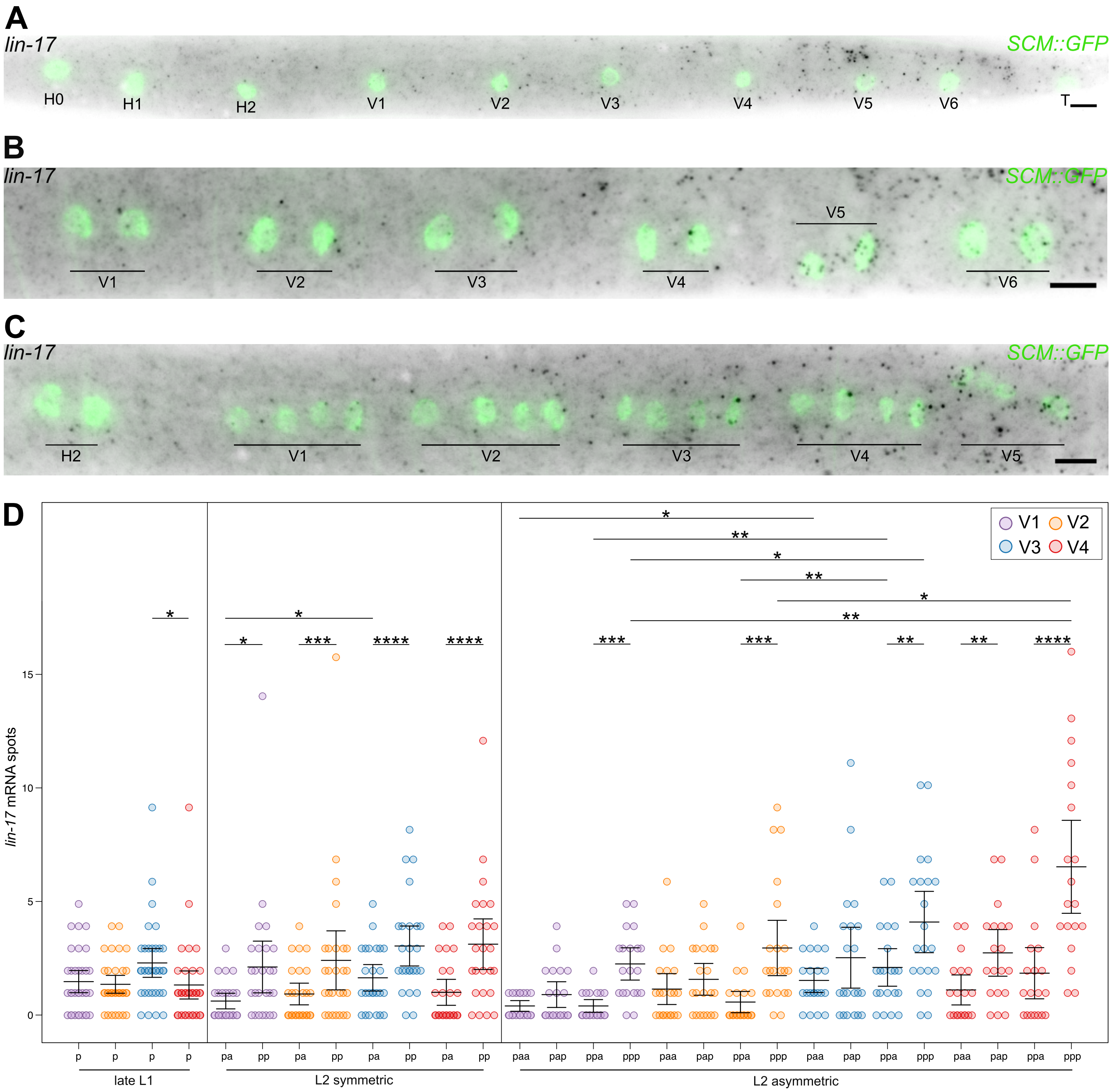

Development • Supplementary information

Fig. S4. Detailed analysis of *lin-17* expression in the epidermis. (A-C) Representative *lin-17* smFISH images at late L1 (A), and following the L2 symmetric (B) and L2 asymmetric (C) divisions. Seam cell nuclei are labelled with *SCM::GFP* and scale bars are 5μm. **(D)** Quantification of *lin-17* mRNA spots in V1-V4 seam cells before (p) and after the L2 symmetric division (pa, pp), and following the L2 asymmetric division (paa, pap, ppa, ppp). Across lineage comparisons were analysed using Friedman tests followed by post hoc Wilcoxon signed-rank tests. Within lineage anterior/posterior comparisons were analysed using paired t-tests or Wilcoxon signed-rank tests, according to normality testing. For simplification, only statistically significant comparisons are indicated and stars represent: **** $p<0.0001$, *** $p<0.001$, ** $p<0.01$ and * $p<0.05$; $19 \leq n \leq 34$ cells per condition. Error bars in D show the mean ± 95% confidence intervals.

Development • Supplementary information

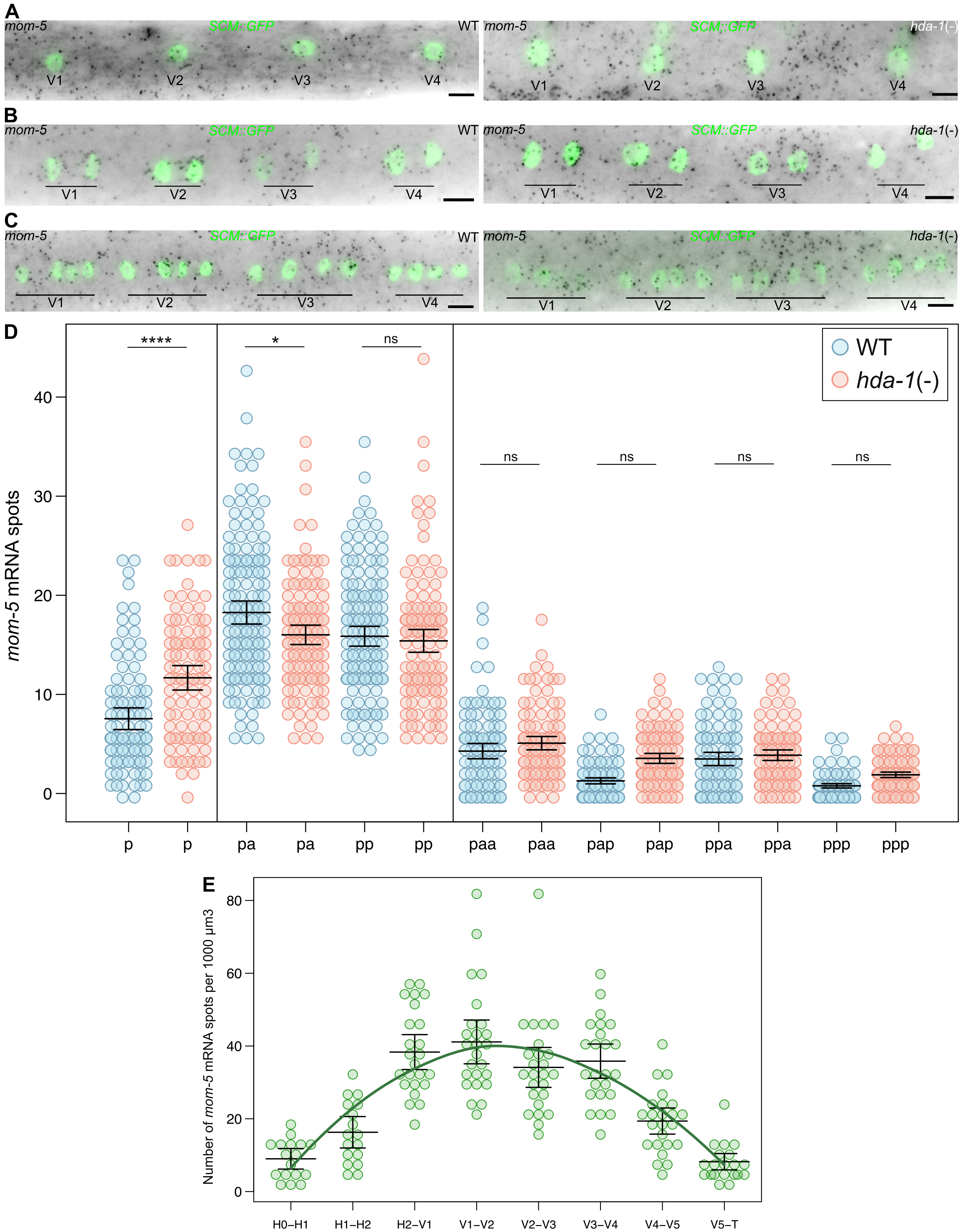
A
B
C
mom-5
SCM::GFP
WT
hda-1(-)
V1
V2
V3
V4
D
****
*
ns
WT
hda-1(-)
mom-5 mRNA spots
0
10
20
30
40
p
pa
pp
paa
pap
ppa
ppp
E
Number of mom-5 mRNA spots per 1000 µm3
0
20
40
60
80
H0–H1
H1–H2
H2–V1
V1–V2
V2–V3
V3–V4
V4–V5
V5–T

Fig. S5. Analysis of *mom-5* expression in *hda-1* mutants. (A-C) Representative *mom-5* smFISH images of WT and tissue-specific *hda-1* mutant animals (*hda-1*(-)) at late L1 (A) and following the L2 symmetric (B) and L2 asymmetric divisions (C). Seam cell nuclei are labelled using *SCM::GFP* and scale bars are 5 μm. **(D)** Quantification of *mom-5* mRNA spots in V1-V4 lineages in WT (blue) and *hda-1* mutant animals (red) at late L1 (p) and following the L2 symmetric (pa, pp) and L2 asymmetric divisions (paa, pap, ppa, ppp); $98 \leq n \leq 99$ (p), $125 \leq n \leq 149$ (pa,pp) and $87 \leq n \leq 102$ cells per condition cells. **(E)** Quantification of *mom-5* smFISH spot density in the lateral epidermis across the anterior-posterior axis; n = 23 animals. The solid line represents a LOESS-smoothed trend of mean mRNA expression across the anterior-posterior axis. Error bars show the mean ± 95% confidence intervals and stars represent: **** p<0.001 and * p<0.05 with a Mann-Whitney test.

Development • Supplementary information

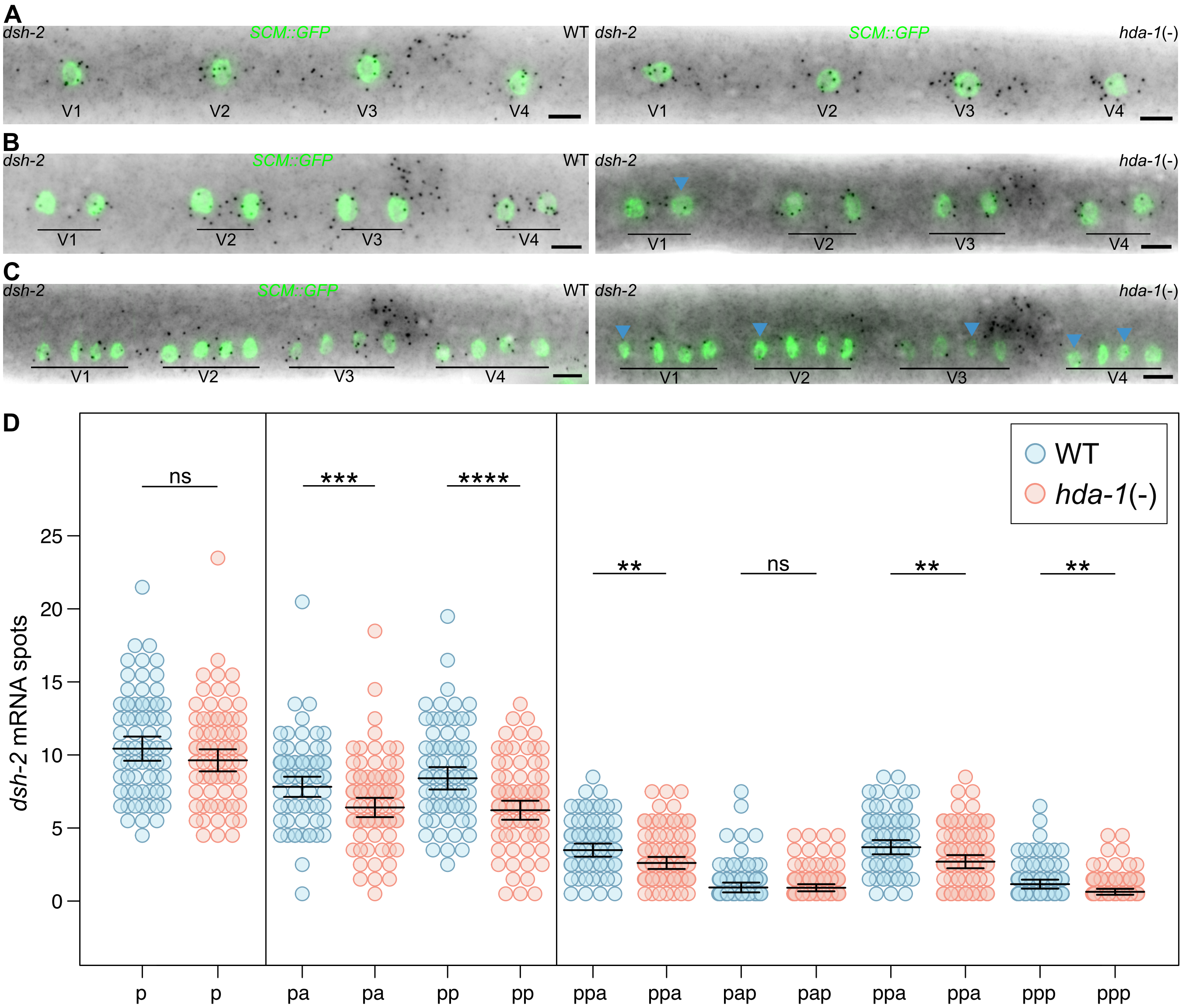

Fig. S6. Analysis of *dsh-2* expression in *hda-1* mutants. (A-C) Representative *dsh- 2* smFISH images of WT and tissue-specific *hda-1* mutant animals (*hda-1*(-)) at late L1 (A) and following the L2 symmetric (B) and L2 asymmetric divisions (C). Seam cell nuclei are labelled using *SCM::GFP* and scale bars are 5 µm. **(D)** Quantification of *dsh-2* mRNA spots in V1-V4 lineages in WT (blue) and *hda-1* mutant animals (red) at late L1 (p) and following the L2 symmetric (pa, pp) and L2 asymmetric divisions (paa, pap, ppa, ppp); $72 \leq n \leq 76$ (p), $72 \leq n \leq 79$ (pa,pp) and $70 \leq n \leq 79$ cells per condition cells. WT and *hda-1*(-) values were compared within each cell type and lineage using a Mann-Whitney test or Welch's t-test following normality testing. Error bars show the mean ± 95% confidence intervals and stars represent ** $p<0.01$, *** $p<0.001$, and **** $p<0.0001$.

Development • Supplementary information

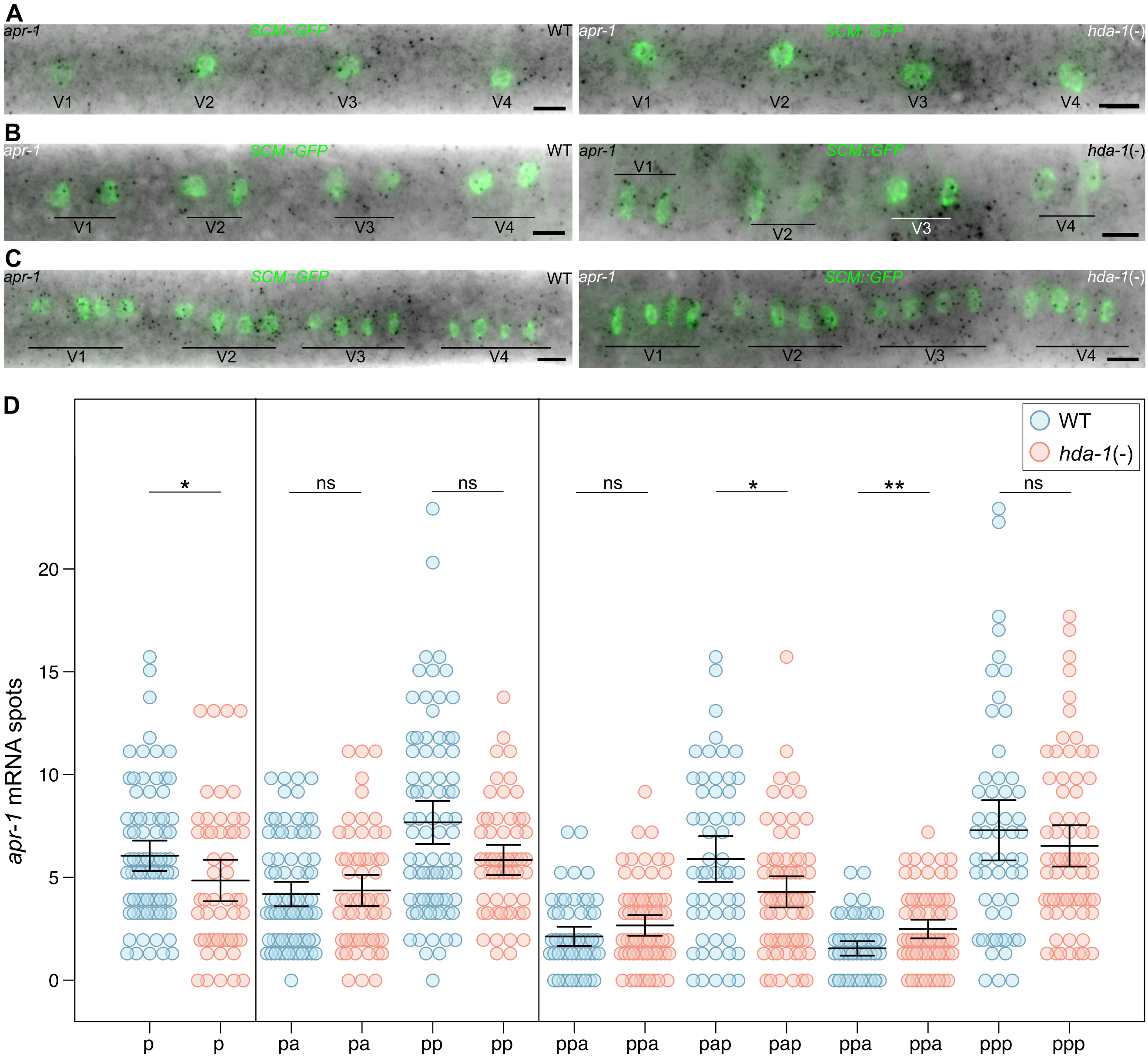

Fig. S7. Analysis of *apr-1* expression in *hda-1* mutants. (A-C) Representative *apr-1* smFISH images of WT and tissue-specific *hda-1* mutant animals (*hda-1*(-)) at late L1 (A) and following the L2 symmetric (B) and L2 asymmetric divisions (C). Seam cell nuclei are labelled using *SCM::GFP* and scale bars are 5 μm. **(D)** Quantification of *apr-1* mRNA spots in V1-V4 lineages in WT (blue) and *hda-1* mutant animals (red) at late L1 (p) and following the L2 symmetric (pa, pp) and L2 asymmetric divisions (paa, pap, ppa, ppp); 52 ≤ n ≤ 80 (p), 58 ≤ n ≤ 80 (pa,pp) and 55 ≤ n ≤ 68 cells per condition cells. Error bars show the mean ± 95% confidence intervals and stars represent: * p<0.05 and ** p<0.01 with a Mann-Whitney test.

Development • Supplementary information

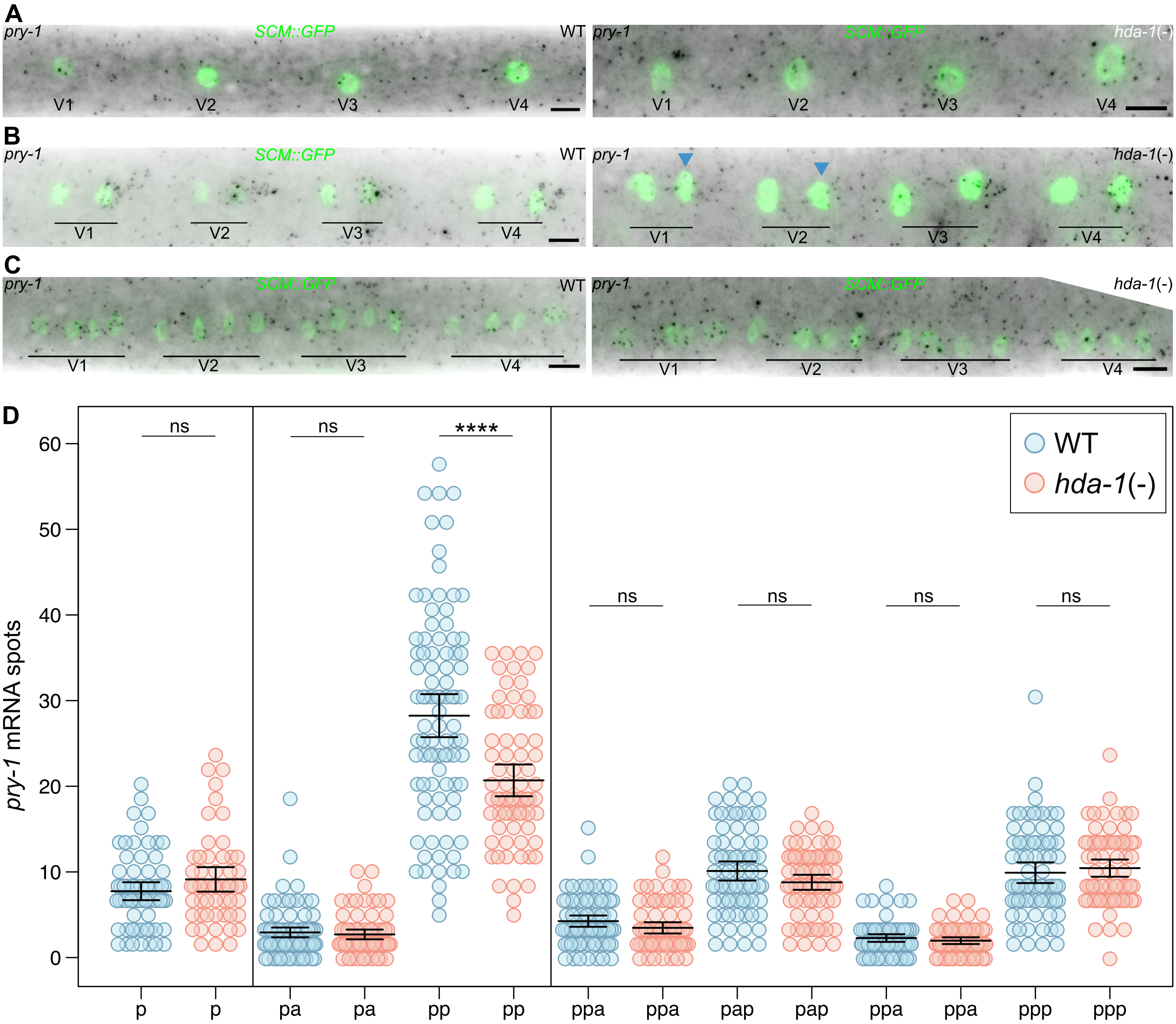

Fig. S8. Analysis of *pry-1* expression in *hda-1* mutants. (A-C) Representative *pry-1* smFISH images of WT and tissue-specific *hda-1* mutant animals (*hda-1*(-)) at late L1 (A) and following the L2 symmetric (B) and L2 asymmetric divisions (C). Seam cell nuclei are labelled using *SCM::GFP* and scale bars are 5 μm. **(D)** Quantification of *pry-1* mRNA spots in V1-V4 lineages in WT (blue) and *hda-1* mutant animals (red) at late L1 (p) and following the L2 symmetric (pa, pp) and L2 asymmetric divisions (paa, pap, ppa, ppp); $54 \leq n \leq 67$ (p), $70 \leq n \leq 91$ (pa,pp) and $70 \leq n \leq 75$ cells per condition cells. WT and *hda-1*(-) values were compared within each cell type and lineage using a Mann-Whitney test or Welch's t-test following normality testing. Error bars show the mean ± 95% confidence intervals and **** represent $p<0.0001$.

Development • Supplementary information

Table S1. HDA-1 TaDa peaks

Available for download at
https://journals.biologists.com/dev/article-lookup/doi/10.1242/dev.205658#supplementary-data

Table S2. Genes corresponding to HDA-1 TaDa peaks and overlap with seam cell expressed genes and known regulators.

Available for download at
https://journals.biologists.com/dev/article-lookup/doi/10.1242/dev.205658#supplementary-data

Table S3. Strains, smFISH probes, vectors and oligos used in this study.

Available for download at
https://journals.biologists.com/dev/article-lookup/doi/10.1242/dev.205658#supplementary-data

Table S4. Extended statistical analysis for all datasets presented in this study.

Available for download at
https://journals.biologists.com/dev/article-lookup/doi/10.1242/dev.205658#supplementary-data
