# Supplementary material for: HDAC1/2-mediated repression of Wnt receptor expression orients asymmetric division polarity in Caenorhabditis elegans
Source: Development. 2026 Sep 3;153(17):dev205658. doi: 10.1242/dev.205658 (PMC13615694; doi:10.1242/dev.205658)
Supplement: Supplementary file 2 [file develop-153-205658_review_history.pdf]

HDAC1/2-mediated repression of Wnt receptor expression orients asymmetric division polarity in Caenorhabditis elegans

Mar Ferrando-Marco, Shuxiao Lin, Beatriz Garcia del Valle, Mark Hintze, Lucy Narunsky, Junyue Huang, Shannon Edwards and Michalis Barkoulas

DOI: 10.1242/dev.205658

Editor: Swathi Arur

Review timeline

Original submission:	10 March 2026
Editorial decision:	24 April 2026
First revision received:	13 July 2026
Accepted:	27 July 2026

Original submission

First decision letter

MS ID#: dev.205658

MS TITLE: HDAC1/2-mediated repression of Wnt receptor expression orients asymmetric division polarity in C. elegans.

AUTHORS: Mar Ferrando-Marco, Shuxiao Lin, Beatriz Garcia del Valle, Mark Hintze, Lucy Narunsky, Junyue Huang, Shannon Edwards and Michalis Barkoulas

Dear Dr Barkoulas,

I have now received all the referees' reports on the above manuscript, and have reached a decision. The referees' comments are appended below.

As you will see, the referees express considerable interest in your work, and provide recommendations on further improving the rigor and clarity of the manuscript, with Reviewer 1 providing recommendations for additional experiments which can further bolster the central conclusions. If you are able to revise the manuscript along the lines suggested, which may involve further experiments, I will be happy to receive a revised version of the manuscript. Your revised paper will be re-reviewed by one or more of the original referees, and acceptance of your manuscript will depend on your addressing satisfactorily the reviewers' major concerns.

Please attend to all of the reviewers' comments and ensure that you upload both a 'clean' version of your Word file, along with a highlighted version clearly showing where you have made changes in the revised manuscript. Please avoid using 'Tracked changes' in Word files as these are lost in PDF conversion. I should be grateful if you would also provide a point-by-point response detailing how you have dealt with the points raised by the reviewers in the 'Response to Reviewers' box. If you do not agree with any of their criticisms or suggestions please explain clearly why this is so.

Reviewer 1

SUMMARY OF THE ADVANCE MADE IN THIS PAPER AND ITS POTENTIAL SIGNIFICANCE TO THE FIELD

© 2026. Published by The Company of Biologists under the terms of the Creative Commons Attribution License (https://creativecommons.org/licenses/by/4.0/).

The manuscript by Ferrando-Marco investigates the role of the histone deacetylase HDA-1 (HDAC) in asymmetric divisions of the C. elegans seam cell lineage. The authors first carefully examine the RNA and protein levels of HDA-1, showing mostly uniform expression with some enrichment in posterior daughter cells following the asymmetric division in the L2 stage. Hda-1 mutants show polarity reversals and additional seam patterning defects. To identify what targets of HDA-1 might be responsible, the authors perform DamID, eventually zooming in on the Wnt receptors LIN-17 and CAM-1. The authors show that the mRNAs are asymmetrically distributed between daughter cells, and moreover normally expressed in opposing gradients. In hda-1 mutants overall levels are increased and the asymmetry and gradient are disrupted. The data are a very thorough characterization of the effects of HDA-1 loss on seam polarity.

SUGGESTIONS TO AUTHORS

The experiments are overall very well performed and thorough, using quantifications of mRNA levels by single-molecule FISH, GFP protein fusions, and DamID. However, the conclusions that are drawn are currently too strong for the data. From the abstract: "Our findings establish a direct link between histone deacetylase activity and Wnt receptor expression and support a model in which graded expression of Wnt receptors provides positional cues that orient asymmetric division polarity in response to Wnt signals." In the discussion, this is strengthened to: "HDA-1 regulates asymmetric cell division polarity in the C. elegans epidermal seam cells by repressing the graded expression of the Wnt receptors lin-17 and cam-1"

The first part of this conclusion is technically true: the loss of hda-1 indeed alters the expression of lin-17 and cam-1, and in polarity defects. However, HDA-1 regulates hundreds or thousands of genes, and the increased lin-17 and cam-1 defects may be a side effect of some general regulatory mechanism rather than a specific role for HDA-1 in asymmetric cell division. In particular since hda-1 expression levels are quite uniform. One way to strengthen this aspect would be to show that the phenotype really is due to lin-17 and cam-1 misregulation by restoring their normal expression levels. Showing that lin-17 or cam-1 overexpression can mimic the phenotype is only one side of the coin. This could be attempted with partial RNAi or use of heterozygous mutant backgrounds.

The second part of the conclusion is that HDA-1 regulates the graded expression of lin-17 and cam-1 to control seam polarity. However, the data on the role of the gradients is quite minimal. In the supplemental data, the authors nicely plot the wild-type gradients in figures S3/S4 E, which is an interesting new finding. But for the cam-1 mutants, the main figures show only the ratio between V1 and V4. Why is the full gradient not shown here and is a simplified representation used? To begin to believe that gradient disruption is the cause of the phenotype, this disruption should be more clearly shown.

Even if the gradient is severely disrupted, we still cannot know that it is the disruption of the gradient that causes the phenotypes rather than overexpression by itself. For that, the authors should devise a way where a gradient can be formed or not formed with similar overall expression levels. Alternatively, the authors should be more open about alternative models and limitation of the study in their discussion.

In addition to these overall comments, the authors should carefully re-evaluate the statistics approaches. Multiple figures contain multi-group comparisons for which a t-test is not appropriate. Two example: In Figure 1B, multiple genotypes are compared to a control. Authors should check for normality and variance, and then either use a one-way ANOVA + Dunnett's post hoc test, or Kruskal-Wallis + Dunn's test. In Figure 3D, the situation is more complex, as there are two variables at play: genotype and anterior/posterior. Here, a two-way ANOVA might be more appropriate (again with proper normality and variance tests, and with appropriate follow-up tests).

In Figure 3D, F, H, the lack of appropriate statistics means that the statement in line 207/208 that "molecular asymmetry between daughter cells was significantly reduced in hda-1 mutants" is not supported. A two-way ANOVA should be used to ask the question: does the difference between anterior and posterior depend on the genotype. The current t-tests are not appropriate and do not address that question. Even better would have been to measure the difference between pairs of cells rather than using a group average.

© 2026. Published by The Company of Biologists under the terms of the Creative Commons Attribution License (https://creativecommons.org/licenses/by/4.0/).

Reviewer 2

SUMMARY OF THE ADVANCE MADE IN THIS PAPER AND ITS POTENTIAL SIGNIFICANCE TO THE FIELD

The ms by Ferrando-Marco et al. reports on how a histone deacetylase, HDA-1, fine-tunes seam cells polarisation by negatively regulating the expression of Wnt pathway components, using the seam cells lineage in live C. elegans as a model system.

First, Ferrando-Marco et al. undertook an extended characterisation of hda-1 expression and the phenotypic consequences of its loss. Since hda-1 general loss is lethal, they developed a useful Cre-Lox tool to inactivate it in their tissue of interest. In particular, using several markers throughout the study, they show that A-P polarity is reversed in some seam cells upon loss of hda-1. Second, this study extends our fine-grained knowledge of Wnt pathway component A-P expression, at the single cell level in a quantitative manner, highlighting antagonistic patterns and dynamics for two Wnt receptors. In addition, using a cell-specific profiling methods, the authors reveal the regions bound by HDA-1 in seam cells, and show that this binding translates into negative regulation of Wnt receptor and co-receptors lin-17 and cam-1 transcription in particular. It is interesting to note that hda-1 loss, while affecting the A-P gradient of these receptors, does not greatly impact their expression dynamics. They nicely further showed that uniform A-P overexpression of these receptors in seam cells phenocopies loss of hda-1, linking polarity defects and A-P Wnt receptor gradients.

The experiments are well conceived and controlled. The data are robust, presented clearly. and support the main conclusions of the manuscript. They support a model in which spatial control of Wnt signal is exerted at the level of Wnt receptors - rather than downstream pathway components, at least in subsets of seam cells, and where their expression territories are fine-tuned through the activity of hda-1. Further work will be needed to decipher which of the numerous HDA-1-containing complexes is involved. Overall, this study brings new knowledge, complementing a large body of work, in our understanding of how polarity orientation between daughter cells is established in a field of cells.

I have only a few comments and questions that should be addressed before publication.

SUGGESTIONS TO AUTHORS

* Fig 1 D-F & S1B : in absence of a membrane staining, how can you ensure that the spot seen are located in seam cells ? If no specific method has been used, it would make sense to redo these experiments using the ajm-1 strain used in Fig 1 H, I, J. It is particularly true for the control experiment depicted in S1B.

* Fig 2D: Could the authors please specify (in the legend) if the % defect represents the total # of instances of the defect, or # of animals showing at least one instance of the defect? (This is not in the M&M either)

* Fig 3C, E: It is unclear, from the figures, if nhr-73 pattern is affected in V1 lineage? While it is in G, suggesting a discrepancy between the eff-1 reversal/polarity reversal specifically in V2 and asymmetric expression of other genes. Could the authors comment?

* L223, 584: how was the "TSS" determined? Does this correspond to the predicted ATG or is another annotation (Serizay et al?) used? This is not in the M&M either.

* L23230-233: please provide in table S2 the full list of candidate "seam cell" genes list aggregated from the literature (RNA seq + home-curated). As is, only the total # of genes (6041) used for comparison with the TaDa peaks is indicated, not the genes themselves, and the resulting 1659 common genes.

* Tables S1, S2: It is not entirely clear (and logical) to me why only the 1,886 statistically significant peaks (FDR < 0.05) are shown in table S1, while (all ?) 3983 peaks are shown in table S2 and are used for comparison with the candidate seam cell transcriptome ? One would have

© 2026. Published by The Company of Biologists under the terms of the Creative Commons Attribution License (https://creativecommons.org/licenses/by/4.0/).

expected to compare only the significant peaks/genes with the seam cell transcriptome -and the first table to correspond to the raw data.
I would have liked to see :
- all peaks (3983 bound regions as in table S2?) with the associated FDR ;
- all peaks annotations to the closest gene (3196);
- the ? genes with a significant peak (FDR < 0.05) (1886 total peaks, unclear how many corresponding genes);
- the comparison between these genes with a significant peak and the seam cell transcriptome.
Could the authors provide these analyses? Does this significantly change the final list of common genes, and if yes, could the authors comment ?
Also, could the author indicate and comment on, for the 1659 common genes that were found (or the new number in point 4) above), where HDA-1 is bound? Could this help identify which of the many known complexes including HDA-1 could be involved here?

* Fig S2F, L242: apr-1 is actually not in the list of shared genes between the TaDa genes and the seam cell regulators provided by table S2 (but is twice in the seam cell regulator list). Nor is lin-17. Please amend. By contrast, mig-1, dsh-1, vang-1, all Wnt-related are and could be highlighted as well. apr-1: whether in the TaDa list or not, looking at all Wnt-related factors involved in seam cell development, makes perfect sense.
Could the authors justify the focus on lin-17 (rather than mig-1 for instance)?

* Fig S3E: could the authors specify the stage used?

* L363-370: It would have been nice to know how loss of the hox genes bound by HDA-1 in seam cells affect Wnt receptor expression.

* L 429-430: hda-1 has been described as a component of many complexes in C. elegans and vertebrates. Please alter the sentences: SIN3, NuRD, MEC plus SMRT/NCoR2, NCor/NCoR1, CoREST, MiDAC, MIER, RERE

* L576: replace "layering" by "laying"

First revision

Author response to reviewers' comments

We thank both reviewers for providing constructive feedback to help us strengthen our manuscript. Here is our point-by-point response to all comments.

# Reviewer 1:
The manuscript by Ferrando-Marco investigates the role of the histone deacetylase HDA-1 (HDAC) in asymmetric divisions of the C. elegans seam cell lineage. The authors first carefully examine the RNA and protein levels of HDA-1, showing mostly uniform expression with some enrichment in posterior daughter cells following the asymmetric division in the L2 stage. Hda-1 mutants show polarity reversals and additional seam patterning defects. To identify what targets of HDA-1 might be responsible, the authors perform DamID, eventually zooming in on the Wnt receptors LIN-17 and CAM-1. The authors show that the mRNAs are asymmetrically distributed between daughter cells, and moreover normally expressed in opposing gradients. In hda-1 mutants overall levels are increased and the asymmetry and gradient are disrupted. The data are a very thorough characterization of the effects of HDA-1 loss on seam polarity.
We thank the reviewer for taking the time to provide feedback.

The experiments are overall very well performed and thorough, using quantifications of mRNA levels by single-molecule FISH, GFP protein fusions, and DamID. However, the conclusions that are drawn are currently too strong for the data. From the abstract: "Our findings establish a

© 2026. Published by The Company of Biologists under the terms of the Creative Commons Attribution License (https://creativecommons.org/licenses/by/4.0/).

direct link between histone deacetylase activity and Wnt receptor expression and support a model in which graded expression of Wnt receptors provides positional cues that orient asymmetric division polarity in response to Wnt signals." In the discussion, this is strengthened to: "HDA-1 regulates asymmetric cell division polarity in the C. elegans epidermal seam cells by repressing the graded expression of the Wnt receptors lin-17 and cam-1"
Thank you. We toned down these statements and revised the abstract to 180 words in accordance with the editorial instructions.

The first part of this conclusion is technically true: the loss of hda-1 indeed alters the expression of lin-17 and cam-1, and in polarity defects. However, HDA-1 regulates hundreds or thousands of genes, and the increased lin-17 and cam-1 defects may be a side effect of some general regulatory mechanism rather than a specific role for HDA-1 in asymmetric cell division. In particular since hda-1 expression levels are quite uniform. One way to strengthen this aspect would be to show that the phenotype really is due to lin-17 and cam-1 misregulation by restoring their normal expression levels. Showing that lin-17 or cam-1 overexpression can mimic the phenotype is only one side of the coin. This could be attempted with partial RNAi or use of heterozygous mutant backgrounds.
Regarding the first point, we agree with the reviewer that demonstrating suppression of the *hda-1* polarity phenotype by reducing *lin-17* or *cam-1* activity would be ideal as complementary to the sufficiency shown by the overexpression experiments. Following the suggestion of the reviewer, we attempted *lin-17* and *cam-1* RNAi in the *hda-1* mutant background, scored by *eff-1* smFISH. However, we found that simply feeding HT115 bacteria partially suppressed the baseline *hda-1*(-) phenotype, limiting the dynamic range available to detect a genetic interaction under these conditions (see data from the *lin-17* RNAi experiment below, n=50 animals). We did not generate *hda-1; lin-17* or *hda-1; cam-1* double mutants because *hda-1* mutants are poorly viable beyond L4. Furthermore, interpretation in both cases is further complicated by the fact that *lin-17* and *cam-1* single mutants have been reported to show some seam cell polarity reversals (PMID: 41507057). Based on the above, we have revised the Discussion to state explicitly this limitation that our data demonstrate sufficiency, not necessity.

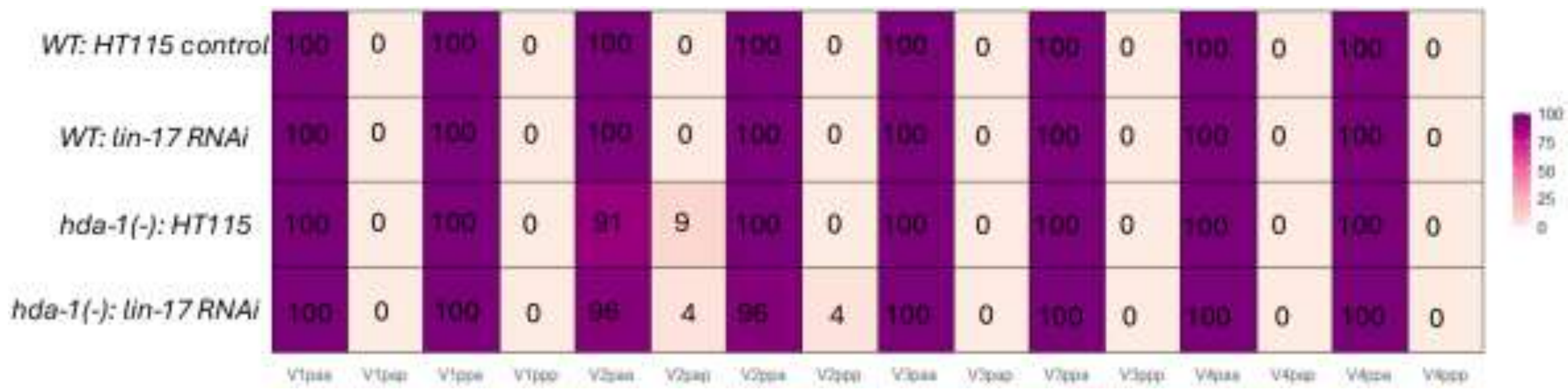

The second part of the conclusion is that HDA-1 regulates the graded expression of lin-17 and cam-1 to control seam polarity. However, the data on the role of the gradients is quite minimal. In the supplemental data, the authors nicely plot the wild-type gradients in figures S3/S4 E, which is an interesting new finding. But for the cam-1 mutants, the main figures show only the ratio between V1 and V4. Why is the full gradient not shown here and is a simplified representation used? To begin to believe that gradient disruption is the cause of the phenotype, this disruption should be more clearly shown.
Thank you for this comment. The normalised V1-V4 difference score shown in the main figures (Figures 4F and 5F) is a simplified visualisation of A-P asymmetry within V1-V4 dividing seam cells. To address whether the broader spatial gradient is disrupted, we followed the reviewer's suggestion and extended the lateral epidermis-wide smFISH quantification we previously showed only for wild-type L1 animals to include *hda-1* mutants. We modelled smFISH spot counts as a function of genotype and AP position using negative binomial regression and tested whether the slope of the gradient differed between genotypes via the genotype × AP- position interaction, using a likelihood-ratio test. For *lin-17*, this analysis showed that the tissue-wide gradient is significantly flattened in *hda-1* mutants. For *cam-1*, by contrast, the tissue-wide gradient is not significantly altered at this stage (while V1-V4 local differences are less pronounced). We have added the new data in the main figures (Figures 4G, 5G) and revised the text in the manuscript to be more precise and distinguish between tissue-wide gradient and lineage-specific differences.

© 2026. Published by The Company of Biologists under the terms of the Creative Commons Attribution License (https://creativecommons.org/licenses/by/4.0/).

Even if the gradient is severely disrupted, we still cannot know that it is the disruption of the gradient that causes the phenotypes rather than overexpression by itself. For that, the authors should devise a way where a gradient can be formed or not formed with similar overall expression levels. Alternatively, the authors should be more open about alternative models and limitation of the study in their discussion.
We thank the reviewer for raising this point. It is certainly interesting but not technically feasible at the moment to flatten the receptor gradient while preserving wild-type expression levels. Therefore, we have made changes in the Discussion acknowledging that our data cannot fully separate disruption of receptor local spatial distribution from increased receptor abundance per se as the cause of the polarity phenotype.

In addition to these overall comments, the authors should carefully re-evaluate the statistics approaches. Multiple figures contain multi-group comparisons for which a t-test is not appropriate. Two example: In Figure 1B, multiple genotypes are compared to a control. Authors should check for normality and variance, and then either use a one-way ANOVA + Dunnett's post hoc test, or Kruskal-Wallis + Dunn's test. In Figure 3D, the situation is more complex, as there are two variables at play: genotype and anterior/posterior. Here, a two- way ANOVA might be more appropriate (again with proper normality and variance tests, and with appropriate follow-up tests). In Figure 3D, F, H, the lack of appropriate statistics means that the statement in line 207/208 that "molecular asymmetry between daughter cells was significantly reduced in hda-1 mutants" is not supported. A two-way ANOVA should be used to ask the question: does the difference between anterior and posterior depend on the genotype. The current t-tests are not appropriate and do not address that question. Even better would have been to measure the difference between pairs of cells rather than using a group average.
We thank the reviewer for this important comment. We have revisited the statistical analyses throughout the manuscript to ensure our statistical approach matches the structure of each dataset and the biological question being asked. For quantitative datasets, we assessed normality using Shapiro-Wilk tests and, where relevant for independent-group comparisons, assessed homogeneity of variance using Levene tests. For datasets involving multiple conditions compared with a shared control, such as Figure 1B, we used one-way ANOVA followed by Dunnett's multiple-comparison test when assumptions were met, or Kruskal-Wallis followed by Dunn's post hoc comparisons when assumptions were not met. For pre-specified WT versus mutant or local pairwise comparisons, we used Welch's t-tests when both groups passed normality, or Mann-Whitney/Wilcoxon rank-sum tests when normality assumptions were not met. For paired anterior/posterior datasets, we calculated within-pair differences, tested these differences for normality, and then used paired t-tests or Wilcoxon signed-rank tests as appropriate. For the more complex anterior/posterior WT versus mutant datasets highlighted by the reviewer, including Figure 3D, F and H, we used a two-way mixed ANOVA, with genotype as a between-pair factor and anterior/posterior position as a within-pair factor. The genotype × position interaction was used as the main test of whether molecular asymmetry depended on genotype. In parallel, and in line with the reviewer's suggestion, we calculated one anterior-posterior difference score per matched pair and compared these difference scores between WT and *hda-1(-)* animals (new panels S1C-E). Categorical heatmap datasets were analysed using Fisher's exact tests. We updated all figure legends, added a statistics section to the materials and methods and included a new supplementary table reporting the tests used, assumption checks and p-values for each analysis.

# Reviewer 2:

The ms by Ferrando-Marco et al. reports on how a histone deacetylase, HDA-1, fine-tunes seam cells polarisation by negatively regulating the expression of Wnt pathway components, using the seam cells lineage in live C. elegans as a model system.

First, Ferrando-Marco et al. undertook an extended characterisation of hda-1 expression and the phenotypic consequences of its loss. Since hda-1 general loss is lethal, they developed a useful Cre-Lox tool to inactivate it in their tissue of interest. In particular, using several markers throughout the study, they show that A-P polarity is reversed in some seam cells upon loss of hda-1. Second, this study extends our fine-grained knowledge of Wnt pathway component A-P expression, at the single cell level in a quantitative manner, highlighting antagonistic patterns and dynamics for two Wnt receptors. In addition, using a cell-specific profiling methods, the authors

© 2026. Published by The Company of Biologists under the terms of the Creative Commons Attribution License (https://creativecommons.org/licenses/by/4.0/).

reveal the regions bound by HDA-1 in seam cells, and show that this binding translates into negative regulation of Wnt receptor and co-receptors lin-17 and cam-1 transcription in particular. It is interesting to note that hda-1 loss, while affecting the A-P gradient of these receptors, does not greatly impact their expression dynamics. They nicely further showed that uniform A-P overexpression of these receptors in seam cells phenocopies loss of hda-1, linking polarity defects and A-P Wnt receptor gradients.

The experiments are well conceived and controlled. The data are robust, presented clearly. and support the main conclusions of the manuscript. They support a model in which spatial control of Wnt signal is exerted at the level of Wnt receptors - rather than downstream pathway components, at least in subsets of seam cells, and where their expression territories are fine-tuned through the activity of hda-1. Further work will be needed to decipher which of the numerous HDA-1-containing complexes is involved. Overall, this study brings new knowledge, complementing a large body of work, in our understanding of how polarity orientation between daughter cells is established in a field of cells.
We thank the reviewer for their feedback.

I have only a few comments and questions that should be addressed before publication.

* Fig 1 D-F & S1B : in absence of a membrane staining, how can you ensure that the spot seen are located in seam cells ? If no specific method has been used, it would make sense to redo these experiments using the ajm-1 strain used in Fig 1 H, I, J. It is particularly true for the control experiment depicted in S1B.
We thank the reviewer for this comment. We have expanded the Materials and Methods to explain how seam-cell regions of interest (ROIs) were defined for quantification. Briefly, seam cells were identified using the SCM::GFP nuclear marker. For quantification, ROIs were manually drawn around each SCM::GFP-positive seam cell nucleus matching the characteristic eye-shaped seam cell morphology. Importantly, ROIs were defined blinded to the smFISH signal, so spot distribution was not used to assign spots to a given cell. Only puncta located within the predefined ROI were included in the quantification. This is something we have a lot of experience in doing in our lab (e.g. PMID: 40026193, PMID: 42281555) and have repeatedly found that seam cell quantifications with or without membrane markers are very comparable in our hands. For Figure S1B, the experiment was intended as a qualitative control to assess the efficiency of seam cell-specific *hda-1* depletion. This panel shows that *hda-1* smFISH signal is strongly reduced around SCM::GFP-positive seam cell nuclei in *hda-1(-)* animals at late L1 while it remains detectable in the surrounding epidermis.

* Fig 2D: Could the authors please specify (in the legend) if the % defect represents the total # of instances of the defect, or # of animals showing at least one instance of the defect? (This is not in the M&M either)
We thank the reviewer for pointing this out. The percentages in Figure 2D represent the percentage of animals showing at least one instance of the indicated defect, rather than the total number of instances of the defect. We have now clarified this in the Figure 2 legend.

* Fig 3C, E: It is unclear, from the figures, if nhr-73 pattern is affected in V1 lineage? While it is in G, suggesting a discrepancy between the eff-1 reversal/polarity reversal specifically in V2 and asymmetric expression of other genes. Could the authors comment?
We thank the reviewer for raising this point. To directly address this comment, we re-analysed the *nhr-73* and *elt-1* data and scored, for each lineage, the percentage of cases in which the normal posterior > anterior relationship was inverted for these two genes (Fig. S1F). This "reversal-like" score was most enriched in the V2 lineage, consistent with the lineage in which *eff-1*-defined polarity reversals are most frequently observed. We also plotted the P-A difference in expression (Fig. S1C-E) for all pairs shown in Figure 3. Finally, we have revised the text to clarify that reduced *nhr-73*/*elt-1* asymmetry alone does not equate to complete fate reversal in the same way that loss of *eff-1* expression does.

* L223, 584: how was the "TSS" determined? Does this correspond to the predicted ATG or is another annotation (Serizay et al?) used? This is not in the M&M either.
We have now clarified this in the Materials and Methods.

© 2026. Published by The Company of Biologists under the terms of the Creative Commons Attribution License (https://creativecommons.org/licenses/by/4.0/).

* L23230-233: please provide in table S2 the full list of candidate "seam cell" genes list aggregated from the literature (RNA seq + home-curated). As is, only the total # of genes (6041) used for comparison with the TaDa peaks is indicated, not the genes themselves, and the resulting 1659 common genes.
Thank you, added.

* Tables S1, S2: It is not entirely clear (and logical) to me why only the 1,886 statistically significant peaks (FDR < 0.05) are shown in table S1, while (all ?) 3983 peaks are shown in table S2 and are used for comparison with the candidate seam cell transcriptome ? One would have expected to compare only the significant peaks/genes with the seam cell transcriptome -and the first table to correspond to the raw data. I would have liked to see :
- all peaks (3983 bound regions as in table S2?) with the associated FDR ;
- all peaks annotations to the closest gene (3196);
- the ? genes with a significant peak (FDR < 0.05) (1886 total peaks, unclear how many corresponding genes);
- the comparison between these genes with a significant peak and the seam cell transcriptome.

Could the authors provide these analyses? Does this significantly change the final list of common genes, and if yes, could the authors comment ?
We thank the reviewer for raising this point, which was indeed unclear in the previous version of the manuscript. Table S1 lists the 1,886 statistically significant peaks (FDR < 0.05) identified by the TaDa pipeline. This is a non-redundant list containing one peak per row. Table S2 is a peak-to-gene annotation table, not a list of 3,983 peaks. Because a single peak can be annotated to more than one nearby gene, the same peak (or gene) can appear on multiple rows, therefore the 3,983 rows in Table S2 correspond to 1,831 unique peaks and 3,195 unique genes. All 1,831 unique peaks fall within the 1,886 significant peaks reported in Table S1. None of the peaks used for gene annotation or for the seam-cell transcriptome comparison are non-significant (the remaining 55 significant peaks did not have any gene within the annotation window and are therefore absent from Table S2). We confirm that the comparison with the seam-cell transcriptome (Cao et al., 2017) was performed using exactly this set of genes linked to significant peaks. We have added a header to Table 2 to clarify this point.

Also, could the author indicate and comment on, for the 1659 common genes that were found (or the new number in point 4) above), where HDA-1 is bound? Could this help identify which of the many known complexes including HDA-1 could be involved here?
For the first point, see plot below, which looks quite similar to what is shown in Figure S2C for all genes containing peaks. For the second part, we compared HDA-1 peaks with available ChIP-seq datasets and there is some overlap especially with NuRD complex components. For example, 40% of 1886 TaDa peaks overlap with HDA-1 ChIP-seq peaks (L3), 20% with MEP-1 peaks (L4), 14% with LIN-40 peaks (YA), 6% with SPR-1 (L4) and 3% with SPR-3 (L4). However, these ChIP-seq datasets contain very different number of peaks and reflect different stages / tissues (TaDa captures mainly targets in the epidermis) making these comparisons difficult to be very informative at this stage. Future work and ideally more comparable datasets will be required to dissect the complex involved in the case of seam cell polarity reversals.

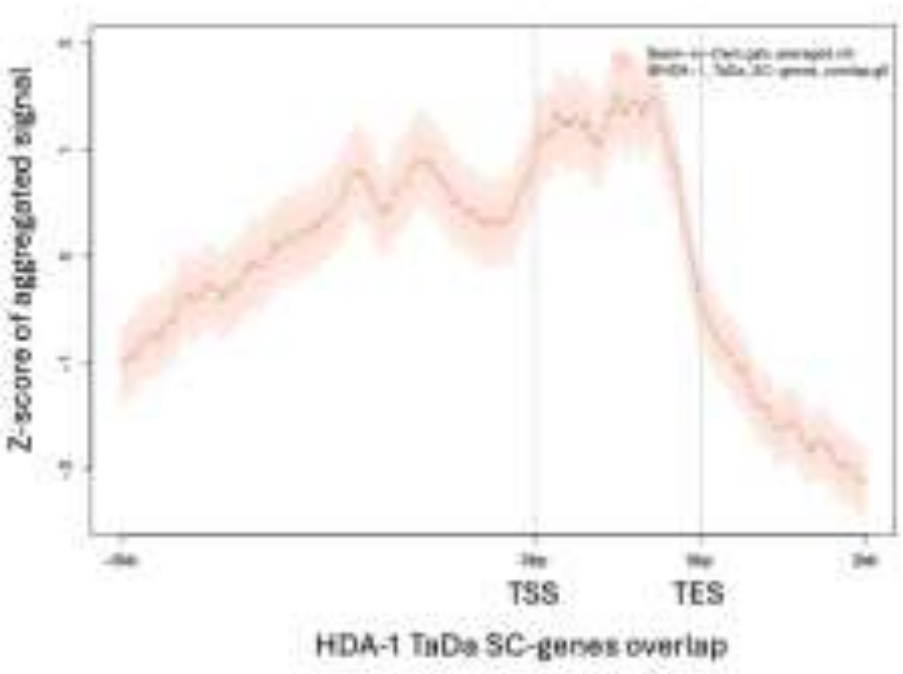

* Fig S2F, L242: apr-1 is actually not in the list of shared genes between the TaDa genes and the seam cell regulators provided by table S2 (but is twice in the seam cell regulator list). Nor is lin-

© 2026. Published by The Company of Biologists under the terms of the Creative Commons Attribution License (https://creativecommons.org/licenses/by/4.0/).

17. Please amend. By contrast, mig-1, dsh-1, vang-1, all Wnt-related are and could be highlighted as well. apr-1: whether in the TaDa list or not, looking at all Wnt-related factors involved in seam cell development, makes perfect sense.
Could the authors justify the focus on lin-17 (rather than mig-1 for instance)?
We thank the reviewer for noticing this error. We have corrected it in Table S2 so *apr-1* is now included among the genes shared between the TaDa targets and seam-cell regulators. We have also re-checked the table and confirm *lin-17* was already correctly listed in this overlap. We apologise for any lack of clarity in Table S2, which should now be resolved with the revised table header (see response above). Regarding the choice to focus smFISH validation on *cam-1*, *lin-17*, *mom-5*, *dsh-2*, *apr-1* and *pry-1*, this was guided by what was already known in the literature for the phenotype of interest. For example, Yamamoto et al. (PMID: 22022276) showed that LIN-17, MOM-5 and CAM-1 are the principal Wnt receptors controlling seam cell polarity, whereas MIG-1 is dispensable in the presence of the main receptors. Furthermore, seam cell polarity is normal in *vang-1* mutants (PMID: 22022276). We have added a sentence to the text clarifying our rationale.

* Fig S3E: could the authors specify the stage used?
Added the information to the figure legend.

* L363-370: It would have been nice to know how loss of the hox genes bound by HDA-1 in seam cells affect Wnt receptor expression.
We agree with the reviewer that this would be interesting point to explore further. As a preliminary step, we examined *cam-1* expression by smFISH in *lin-39* mutants, since *lin-39* is among the Hox genes bound by HDA-1 in our TaDa dataset. At the late L1 stage, *cam-1* levels were comparable between WT and *lin-39(-)* across all seam cell lineages. Following the L2 asymmetric division, *cam-1* was significantly increased in *lin-39(-)* but only in a subset of lineages. These results are still preliminary but suggest that Hox gene loss may affect Wnt receptor expression. We plan to extend this analysis in the future to other Hox genes and Wnt receptors identified in our dataset to investigate a potential role of Hox genes in the control of seam cell polarity and its link (if any) to HDA-1 regulation of Wnt receptor expression.

Late L1 stage

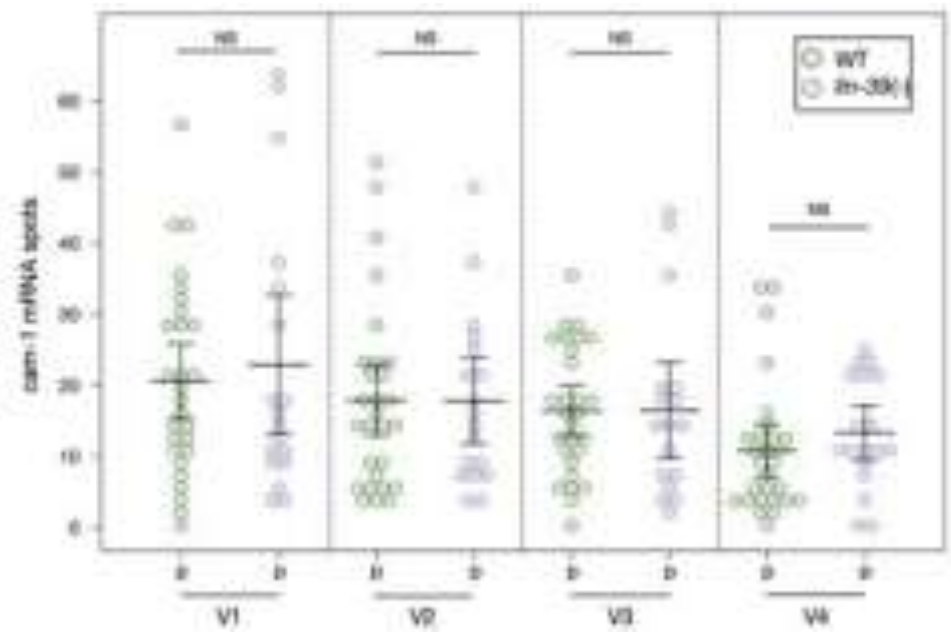

Late L2 stage

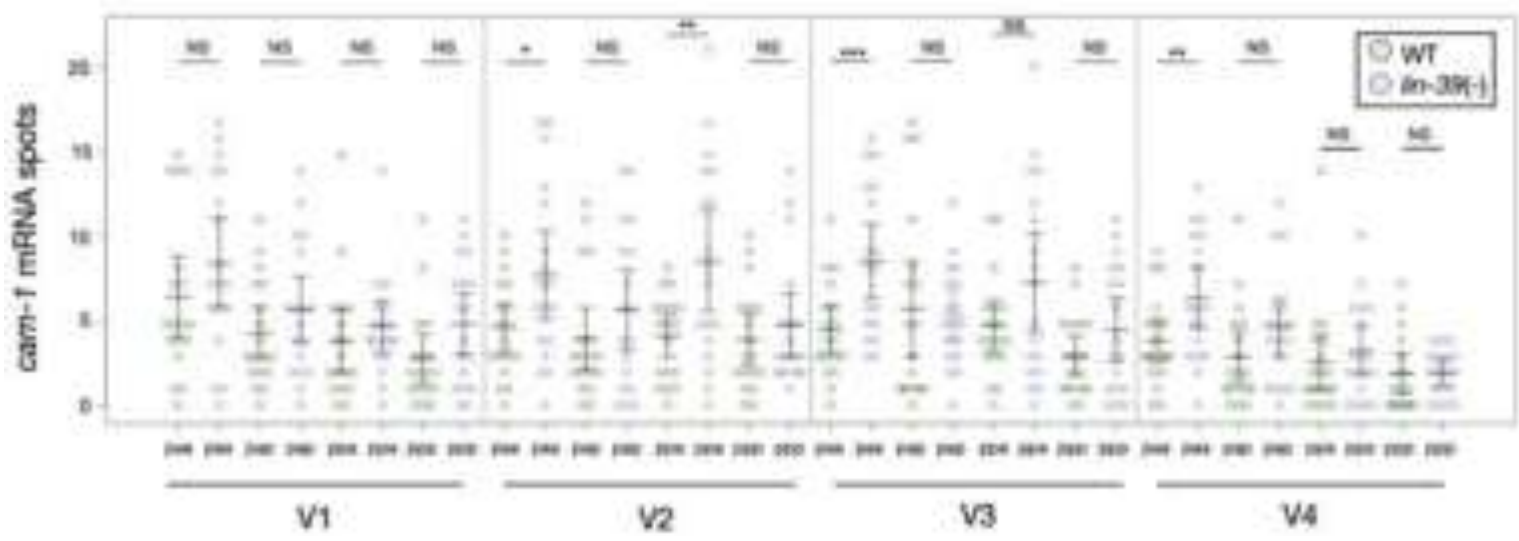

* L 429-430: hda-1 has been described as a component of many complexes in C. elegans and vertebrates. Please alter the sentences: SIN3, NuRD, MEC plus SMRT/NCoR2, NCor/NCoR1, CoREST, MiDAC, MIER, RERE.
Thank you, added

* L576: replace "layering" by "laying"
Corrected

© 2026. Published by The Company of Biologists under the terms of the Creative Commons Attribution License (https://creativecommons.org/licenses/by/4.0/).

Second decision letter

MS ID#: dev.205658R1

MS TITLE: HDAC1/2-mediated repression of Wnt receptor expression orients asymmetric division polarity in C. elegans.

AUTHORS: Mar Ferrando-Marco, Shuxiao Lin, Beatriz Garcia del Valle, Mark Hintze, Lucy Narunsky, Junyue Huang, Shannon Edwards and Michalis Barkoulas

Dear Dr Barkoulas,

I am happy to tell you that your manuscript has been accepted for publication in Development, pending our standard publication integrity checks.

Reviewer 1

SUMMARY OF THE ADVANCE MADE IN THIS PAPER AND ITS POTENTIAL SIGNIFICANCE TO THE FIELD

The manuscript by Ferrando-Marco investigates the role of the histone deacetylase HDA-1 (HDAC) in asymmetric divisions of the C. elegans seam cell lineage. Asymmetric cell divisions are a cornerstone of multicellular development and the seam cell lineage has been used for decades as a model system. Past studies have led to extensive knowledge of Wnt asymmetry pathways, but the full set of mechanisms controlling asymmetric divisions of this stem cell-like lineage are not known. The novelty of this manuscript lies in the linking of HDA-1 to the generation of gradients of the Wnt receptors lin-17/Frizzled and cam-1/Ror. Thus, histone deacetylase activity may be directly involved in generating the cues that lead to asymmetric cell division. It will be interesting to determine if histone modification in other systems similarly can be involved in asymmetric cell divisions.

SUGGESTIONS TO AUTHORS

I thank the authors for the changes they have made to the manuscript. I appreciate that my suggestions were technically difficult to implement and am fine with the alternative of toning down the conclusions and expanding explanations of limitations of the study.

© 2026. Published by The Company of Biologists under the terms of the Creative Commons Attribution License (https://creativecommons.org/licenses/by/4.0/).
